# Some Remarks on Hernia

**Published:** 1908-12-26

**Authors:** 


### SOME REMARKS ON HERNIA.

### VIII.—SOME LESS COMMON FORMS OF HERNIA.

A large group of conditions are classed under the somewhat vague heading of ventral hernia. Roughly, the term is used to indicate any protrusion of the contents of the abdomen through a weak spot in the abdominal parietes other than the inguinal or femoral openings.

The variety most frequently seen is the post-operative, which is caused by a giving way of the line of union at the site of an old incision. The muscular edges become separated and the scar stretched. As a result of this a swelling makes its appearance, owing to the protrusion of abdominal contents, intestine, or omentum, or both. Such a condition rarely gives rise to any symptoms of a serious nature. The patient complains of the presence of an abnormal swelling, which shows a tendency to increase in size progressively. Obstruction of such a hernia and, *a fortiori*, strangulation are rare features. Post-operative hernia occurring in the upper segment of the abdomen is not at all common. This is only what might be expected, since hardly any pressure is exerted on the parietes in this position. Even when the patient is in the upright attitude it all comes on the abdominal wall below the umbilicus. For this reason a hernia hardly ever makes its appearance through a wound in the epigastric or lumbar regions, even in cases in which the original wound did not heal by first intention, or in which it was found necessary to insert a tube or a gauze drain; as, for instance, in pyonephrosis or perforated gastric ulcer. In such cases the normal abdominal tension, which acts more in a downward than in a forward direction, tends to keep the edges of the wound in apposition rather than to force them apart. So that a linear scar is the rule rather than the exception in these situations.

Below the umbilicus, on the other hand, post-operative hernia occurs with unpleasing frequency. It is, however, not now so common after operations for appendicitis as it used to be before the introduction of the "muscle-splitting" or "gridiron" method. In this an incision is made over McBurney's spot, and the muscles of the abdominal wall, instead of being cut, as used to be the fashion, are divided in the direction of their fibres. This gives a very good exposure, and the strength of the resisting power of the abdominal wall is hardly, if at all, weakened by the proceeding. In any ordinary case of appendicitis it is therefore strongly to be advised; but there are cases in which the surgeon might be handicapped by employing it, as, for instance, in dealing with an appendix abscess in which there is œdema of the tissues of the abdominal wall; and in these the muscles must be cut.

A hernia is also a not uncommon sequela of gynæcological operations in which a median suprapubic incision is necessary. Many methods of sewing up have been suggested with the special object of preventing a subsequent hernia. Much stress is laid on the value of bringing the inner edges of the two recti together so that one overlaps the other; but it would appear that an equally efficient result is produced if the anterior layer of the aponeurosis is carefully sutured.

The treatment of these cases is theoretically simple. An elliptical incision is made which includes the stretched skin and scar tissue covering the wound, and a careful dissection is made until the sac is exposed. This is removed, the intestines returned, and the wound securely sewn up in layers. But in practice there are many difficulties in the operation. In the first place, there are usually many old adhesions, and the intestine may in this way be directly attached to the posterior surface of the thin skin covering the hernia. Great caution must therefore be used in making the incision in case the gut is damaged. Then one is advised to isolate the sac. This is often more easily said than done; and in some cases there is no real peritoneal covering to the hernia, the intestine having made its way through the original aperture before it united. If there is a definite sac at all it is generally very thin, so that it is difficult to recognise and easy to tear; moreover, it is not pedunculated, and cannot be ligatured and removed as in an inguinal hernia, but must be cut away and the resulting aperture in the peritoneum closed with sutures. The abdominal wall is then sewn up in layers. These operations are not always successful: the hernia tends to reappear, sometimes as soon as the patient gets up, but generally, it must be admitted, after a longer interval.

A ventral hernia sometimes makes its appearance quite apart from any operation wound. This is due to a giving way of the linea alba below the umbilicus, the so-called *diastema rectorum*. The great majority of these cases occur in elderly multiparæ, in whom the abdominal wall has been stretched by repeated parturition, so that the normal elastic support of the parietes is lost. The recti are, so to speak, unable to recover their tone, and the result is that they prolapse and fall apart. The condition may also result from the removal of large intra-abdominal tumours, especially in elderly patients, in whom there is absorption of fatty tissue. As might be expected from its causation, this form of hernia is almost exclusively confined to the female sex. In a typical case there is a wide separation between the two recti through which the intestines bulge, covered only by the thinned skin of the abdominal wall. The patients rarely complain of anything beyond the presence of an unsightly swelling. Complications such as strangulation are almost unknown, because of the size of the opening and the laxity of the edges of the aperture. The best treatment is a well-fitting abdominal belt; otherwise to make an elliptical incision, remove the redundant thin skin and unite the edges of the recti firmly with buried silk-worm gut or silk sutures. But it must be remembered that it is quite useless to perform this operation on a married woman who is likely to have any more children, because the condition will certainly recur if she again becomes pregnant. In our opinion a belt is the best treatment in any case, because it provides artificial support, and thus lessens the likelihood of the occurrence of visceroptosis, which is the commonest complication of this disorder.

